# Mapping QTLs for oil traits and eQTLs for oleosin genes in jatropha

**DOI:** 10.1186/1471-2229-11-132

**Published:** 2011-09-29

**Authors:** Peng Liu, Chun Ming Wang, Lei Li, Fei Sun, Peng Liu, Gen Hua Yue

**Affiliations:** 1 Molecular Population Genetics Group, Temasek Life Sciences Laboratory, 1 Research Link, National University of Singapore, 117604 Singapore

## Abstract

**Background:**

The major fatty acids in seed oil of jatropha, a biofuel crop, are palmitic acid (C16:0), stearic acid (C18:0), oleic acid (C18:1) and linoleic acid (C18:2). High oleic acid and total oil content are desirable for jatropha breeding. Until now, little was known about the genetic bases of these oil traits in jatropha. In this study, quantitative trait locus (QTL) and expression QTL analyses were applied to identify genetic factors that are relevant to seed oil traits in jatropha.

**Results:**

Composite interval mapping identified 18 QTL underlying the oil traits. A highly significant QTL *qC18:1-1 *was detected at one end of linkage group (LG) 1 with logarithm of the odd (LOD) 18.4 and percentage of variance explained (PVE) 36.0%. Interestingly, the QTL *qC18:1-1 *overlapped with *qC18:2-1*, controlling oleic acid and linoleic acid compositions. Among the significant QTL controlling total oil content, *qOilC-4 *was mapped on LG4 a relatively high significant level with LOD 5.0 and PVE 11.1%. Meanwhile, oleosins are the major composition in oil body affecting oil traits; we therefore developed SNP markers in three oleosin genes *OleI*, *OleII *and *OleIII*, which were mapped onto the linkage map. *OleI *and *OleIII *were mapped on LG5, closing to QTLs controlling oleic acid and stearic acid. We further determined the expressions of *OleI*, *OleII *and *OleIII *in mature seeds from the QTL mapping population, and detected expression QTLs (eQTLs) of the three genes on LGs 5, 6 and 8 respectively. The eQTL of *OleIII*, *qOleIII-5*, was detected on LG5 with PVE 11.7% and overlapped with QTLs controlling stearic acid and oleic acid, implying a cis- or trans-element for the *OleIII *affecting fatty acid compositions.

**Conclusion:**

We identified 18 QTLs underlying the oil traits and 3 eQTLs of the oleosin acid genes. The QTLs and eQTLs, especially *qC18:1-1*, *qOilC-4 *and *qOleIII-5 *with contribution rates (R^2^) higher than 10%, controlling oleic acid, total oil content and oleosin gene expression respectively, will provide indispensable data for initiating molecular breeding to improve seed oil traits in jatropha, the key crop for biodiesel production.

## Background

*Jatropha curcas *is becoming one of the world's key crops for biodiesel production [1]. Oil containing a high amount of unsaturated fatty acid can find an application as biodiesel feed stock. To make the production of jatropha profitable and sustainable, genetic improvement of oil yield and quality is demanded. However, oil traits cannot be evaluated until the seeds are harvested and analyzed in laboratory, and detailed selective breeding has not been carried out. Meanwhile molecular breeding in jatropha has not been started due to lack of molecular bases of economically important traits such as seed yield, seed oil traits, biotic or abiotic stress resistance.

Most economically important traits are quantitative and determined by many genes and gene complex where are described as quantitative trait loci (QTLs). Traditional methods of genetic improvement of quantitative traits have relied mainly on phenotype and pedigree information [2], which are easily influenced by environmental factors. To conduct marker assisted selection (MAS) for genetic improvement of oil yield and quality in jatropha, the molecular bases of seed oil traits need to be understood by identifying genomic regions that contain favorite loci, i.e. QTL analysis. QTL analysis has been performed to detect the genetic bases of important agronomic or physiological traits, providing valuable information for trait improvement. Genetic markers have made it possible to detect QTLs that are significantly associated with traits, and made selection more effective [3]. Genetic response can be improved by including the QTLs in marker-assisted selection, which is a method of selection that makes use of phenotypic, genotypic and pedigree data [4]. Moreover, MAS for oil traits improvement will be much advantageous compared to traditional breeding because seed oil traits cannot be measured at early stage or in field. The use of DNA markers for selection in jatropha can greatly reduce breeding scale. By using MAS, decisions can be made at the nursery stage, regarding which individuals should be retained as breeding stock, and which should be removed.

To conduct QTL analysis, most appropriate crosses need to be selected to generate sufficient genetic variations both on DNA and phenotype levels. QTL analyses of total oil content have been made in a number of crops, including oilseed rape[5], soybean[6], maize[7], and sunflower[8]. Recent surveys have shown large variations in content and fatty acid composition of seed oil of Arabidopsis, suggesting populations derived from selected crosses will be useful for investigating these traits [9].

Diversity in gene expression is one of the mechanisms underlying phenotypic diversity among individuals and regarded as one of quantitative traits [10]. Analysis of determinants of candidate gene expression not only helps in understanding the mechanisms for phenotypic variation but also provides an approach to improve phenotypes via the modulation of gene expression[10]. With advances in gene expression profiling, an approach named "genetical genomics" has been put forward to identify the determinants of gene expression [11]. This approach treats mRNA expression levels as quantitative traits in a segregating population and maps expression QTL (eQTL) that control expression levels in vivo. For almost any gene analyzed in a segregating population, eQTL analysis can identify the genomic regions influencing its expression level. eQTL that map to the same genetic location as the gene whose transcript is being measured generally indicate the presence of a cis-acting regulatory polymorphism in the gene (cis-eQTL). eQTL that map distant to the location of the gene being assayed most likely identify the location of trans-acting regulators (trans-eQTL) that may control the expression of a number of genes elsewhere in the genome. The genetical genomics approach has been employed for identifying eQTL regulating gene expression [10,12].

Recently, we established a first generation genetic linkage map of jatropha using 506 microsatellite and SNP markers covering 11 linkage groups [13], thus providing a necessary tool for a whole genome scan for QTLs and eQTLs affecting economically important traits including seed oil traits. Among the fatty acid present in the jatropha seed oil, linoleic acid (18:2), oleic acid (18:1), palmitic acid (16:0) and stearic acid (18:0) are dominant compositions. Oleic and linoleic acids are the major constituents of jatropha oil [14]. The breeding goal for jatropha seed oil trait improvement is to increase total oil content and oleic acid, and decrease palmitic content [15]. In this paper, we describe the genetic bases of these seed fatty acid composition and content traits through QTL mapping with a backcrossing population consisting 286 individuals. On the other hand, seed oil is stored in subcellular organelles called oil bodies. Proteome composition of the jatropha oil bodies revealed oleosins as the major component affecting oil traits [16]. Three jatropha oleosin genes, namely *OleI*, *OleII *and *OleIII*, were isolated [17]. Here, we developed SNPs of the three oleosin genes in the QTL mapping population, which were subsequently mapped onto the linkage map. We determined expression variations of the three genes in the QTL mapping population, conducted an eQTL analysis on oleosin gene expressions and provided new information for possible modulation of oleosin genes to improve oil traits in jatropha.

## Results

### Trait analysis

Fatty acid composition, total oil content of jatropha seeds and gene expression levels of oleosin genes were measured in the QTL mapping population. The frequency distributions of the traits showed a continuous distribution (data not shown), revealing complex genetic bases of these traits. As expected for an interspecific cross, distribution of phenotypic values in the progeny showed bi-directional transgressive segregations for all traits (Table 1). C18:1 in *J. curcas *is higher than in *J. integerrima*, while total oil content in *J. integerrima *is 51.04%, much higher than in *J. curcas*. The data implied that *J. integerrima *germplasm could be applied for hybrid breeding to improve agronomic traits such as total oil content.

**Table 1:** Descriptive statistics on phenotype data of the QTL mapping population and parents (*J.curcas *PZM16 and *J. integerrima *S001)

Traits	Mean	SD	Min	Max	PZM16(Mean ± SD)	S001(Mean ± SD)
C16:0 (%)	12.6	2.54	7.95	21.51	18.88 ± 5.7	7.89 ± 0.25
C18:0 (%)	6.28	1.44	2.89	10.42	6.02 ± 1.01	5.13 ± 0.31
C18:1 (%)	37.72	9.41	18.44	61.77	42.42 ± 0.54	30.83 ± 3.64
C18:2 (%)	43.39	9.96	20.57	66.22	32.7 ± 5.23	56.14 ± 3.58
Total oil content (%)	35.4	8	13.3	57.1	30.59 ± 0.70	51.04 ± 2.39
*OleI *expression (ΔΔC_T_)	-0.32	3.63	-9.89	8.97	0	-0.42
*OleII *expression (ΔΔC_T_)	2.13	3.78	-6.03	11.33	0	2.54
*OleIII *expression (ΔΔC_T_)	-3.4	4.05	-12.1	3.63	0	0.98

Correlation analysis among these traits was performed (Table 2). C18:2 showed a significantly negative correlation with C18:1 and C16:0. Especially the C18:1 correlated with C18:2 with a high coefficient -0.962, implying that there could be common genetic factors affecting these two compositions. The expression levels of *OleI*, *OleII *and *OleIII *showed a highly positive correlation with each other. *OleI *expression level was significantly correlated with C16:0. The correlation coefficients between expression levels of oleosin genes and total oil content were low but significant. The significant but low values of correlation coefficients implied genetic bases of fatty acid composition and total oil content were complex, and oleosin genes could be involved the multiple genetic factors affecting these oil traits.

**Table 2:** Correlation coefficients and significance of correlations among fatty acid composition, total oil content, oleosin gene expressions in a jatropha QTL mapping population

Traits	C16:0	C18:0	C18:1	C18:2	Total oil content	*OleI *expression	*OleII expression*
C18:0	-0.147						
C18:1	0.038	0.05					
C18:2	-0.270**	-0.155	-0.962**				
Total oil content	-0.087	-0.02	-0.003	0.028			
*OleI *expression	0.216**	-0.029	-0.013	-0.038	0.161*		
*OleII *expression	0.139	-0.047	-0.074	0.043	0.191*	0.696**	
*OleIII *expression	0.132	-0.005	0.136	-0.157	0.170*	0.790**	0.697**

*P *values are as follows: * *P *< 0.05, ** *P *< 0.01.

### QTL and eQTL mapping

The linkage map, covering 663.0 cM of the genome, converged into 11 linkage groups consisting of 95 DNA markers. The average distance between markers was 7.0 cM. Most of the linkage groups were consistent with those described previously [13].

**Figure 1:**
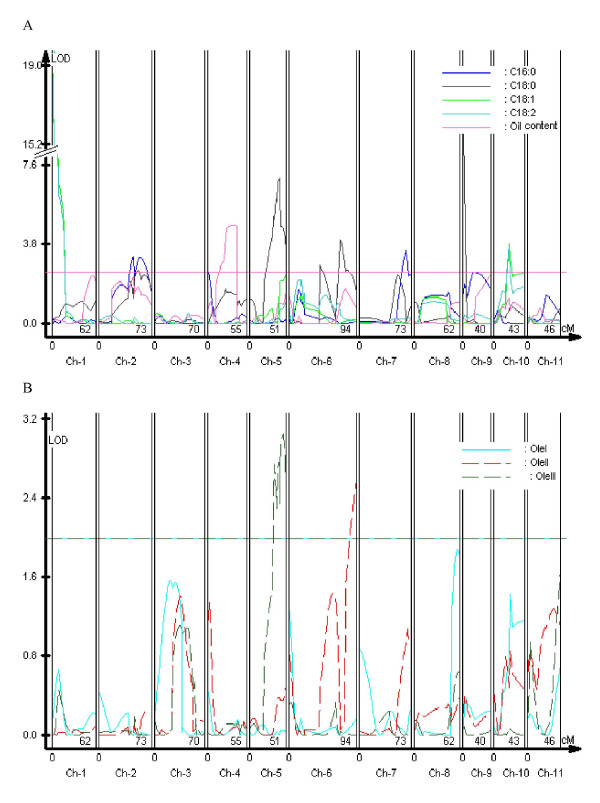
**Whole genome scan for QTL for oil traits and Oleosin gene expression in jatropha**. A QTL scans of oil traits on linkage maps. Horizontal line indicates 5% LOD significance thresholds (2.5) based on permutation. B QTL scans of *OleI*, *OleII *and *OleIII *expressions on linkage maps. Horizontal line indicates LOD significance threshold (2.0).

**Figure 2:**
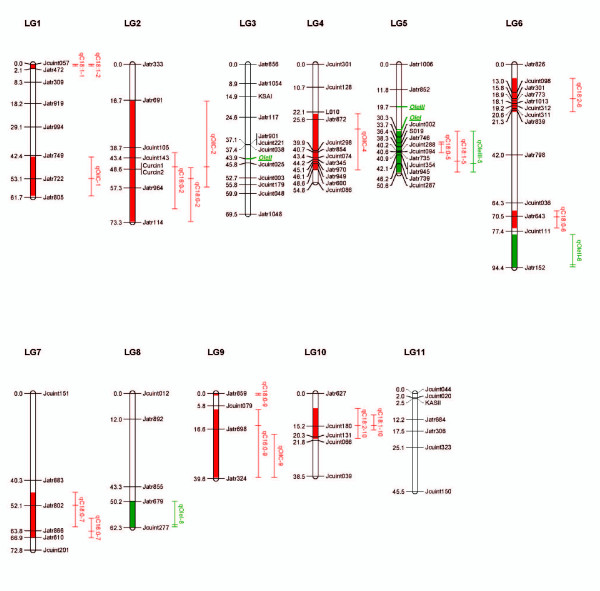
**Summary of QTL (eQTL) locations detected on the genome of jatropha**. QTLs (eQTLs) represented by bars are shown on the left of the linkage groups, close to their corresponding markers. The lengths of the bars are proportional to the confidence intervals of the corresponding QTLs (eQTLs) in which the inner line indicates position of maximum LOD score.

QTL analyses were performed on the means of fatty acid composition, total oil content and expression levels of *OleI*, *OleII *and *OleIII *(Table 3; Figure 1). We detected 18 QTLs and 3 eQTLs for all traits examined. Individual eQTL or QTL were detected with percentage of variation explained (PVE or r^2^) 2.3% to 36.0%, and 5 of them had PVE exceeding 10%. QTLs or eQTLs with positive and negative allelic effects were identified, with a positive effect implying a higher value for the trait conferred by the allele from PZM16 and vice versa (Figure 2).

**Table 3:** QTLs for seed oil traits and eQTLs for *OleI*, *OleII *and *OleIII *expressions in jatropha

Trait	QTL^a^	Linkage	Marker	Position^b^	LOD	R^2 c^	Additive
	(eQTL)	Group		cM	Peak	^(%)^	Effects^d^
*C16:0 (%)*	*qC16:0-2*	2	Jcuint143	47.4	2.6	0.1	1.36
	*qC16:0-7*	7	Jatr802	52.1	3.1	7.4	1.42
	*qC16:0-9*	9	Jatr859	15	2.6	7.2	1.39

*C18:0 (%)*	*qC18:0-2*	2	curcin2	52.6	2.6	5.3	-0.69
	*qC18:0-5*	5	Jatr746	37.3	6.9	13	1.15
	*qC18:0-6*	6	Jcuint036	64	3.9	7.1	-0.84
	*qC18:0-7*	7	Jatr883	40.3	2.3	4	0.59
	*qC18:0-9*	9	Jatr859	0	9.2	17.9	1.26

*C18:1 (%)*	*qC18:1-1*	1	Jcuint057	0	18.4	36	11.69
	*qC18:1-5*	5	Jatr739	45.1	2.3	3.4	-3.77
	*qC18:1-10*	10	Jcuint180	15.2	4	5.9	4.75

*C18:2 (%)*	*qC18:2-1*	1	Jcuint057	0	16.5	34.1	-12.07
	*qC18:2-6*	6	Jatr301	15	2.4	3.8	4.3
	*qC18:2-10*	10	Jcuint180	15.2	3	4.6	-4.4

*Total oil content (%)*	*qOilC-1*	1	Jatr722	55.1	2.3	4.6	-3.72
	*qOilC-2*	2	Jcuint143	47.4	2.5	4.9	-3.74
	*qOilC-4*	4	Jatr872	29.6	5	11.1	-5.56
	*qOilC-9*	9	Jatr698	18.6	2.5	5.2	3.74

*OleI *expression (ΔΔC_T_)	*qOleI-8*	8	Jcuint277	58.2	1.9	5.3	1.71

*OleII *expression (ΔΔC_T_)	*qOleII-6*	6	Jatr152	93.4	2.6	6.4	-2.38

*OleIII *expression (ΔΔC_T_)	*qOleIII-5*	5	Jatr739	46.2	3.1	11.7	-3.06

^a ^QTL (eQTL): starting with "q," followed by an abbreviation of the trait name, the name of the linkage group, and the number of QTLs (eQTLs) affecting the trait on the linkage group. *OleI*, *OleI *expression level; *OleII*, *OleII *expression level; C16:0, C18:0,18:1 and C18:2, fatty acid compositions of palmitic acid (C16:0), stearic acid (C18:0), oleic acid (C18:1) and Linoleic acid (C18:2); OilC: Total oil content

^b ^Position from the first marker on each linkage group.

^c ^Proportion of phenotypic variance (R^2^) explained by a QTL (eQTL).

^d ^Estimated phenotypic effect of substituting *J. integerrima *alleles with *J. curcas *alleles at QTL (eQTL).

### QTLs for fatty acid composition and total oil content

Eighteen QTLs were identified dispersed among all the linkage groups except LGs 3 and 11. A QTL of highly significant effect was determined to be located on LG1 explaining 36% of variation of C18:1 composition, and was found to be associated with C18:2 compositions (Figure 2). Interestingly, another QTL on LG10 explained 5.9% of variation of C18:1 composition was also associated with C18:2 compositions. Higher values for C18:1 were conferred by the allele from PZM16, while higher values for C18:2 from Hybrid CI7041.

Four QTLs were detected underlying total oil content. At the three QTLs on LGs 1, 2 and 4 respectively, the alleles from hybrid CI7041 contributed high total oil content. The most effective QTL was spotted on LG4 explaining 11.1% of the variation, whose higher value for total oil content was conferred by the allele from hybrid CI7041.

### Favorite allele's effects

**Figure 3:**
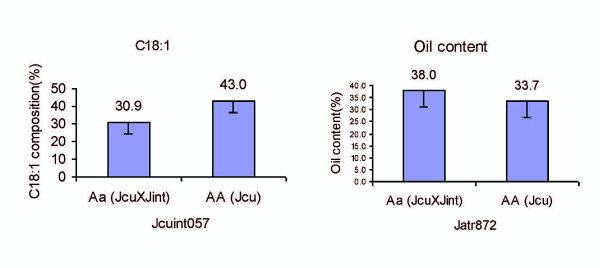
**C18:1 composition (left) and total oil content (right) of plants with different genotypes**. Favorite alleles for C18:1 composition are AA from *J. curcas*, and those for total oil content are Aa from hybrid of *J. integerrima *and *J. curcas *(right).

There were strong QTLs for C18:1 and total oil content detected on LGs 1 and 4, respectively. Mean phenotypic values of each trait were calculated for those progeny with the alternate alleles of the microsatellite markers, inherited from the *J. integerrima *(aa) or *J. curcas *(AA). A two-way ANOVA was performed on the progeny using two allelic combinations (AA, Aa) from markers linked to QTLs in order to investigate associations between phenotypic traits and genotypes of the QTLs. The phenotype values of each allelic combination of the QTLs are listed in Figure 3. Significant differences of phenotype means among different allelic combinations were identified, revealing the effects of alternative alleles inherited from the parents.

Progenies with AA genotype at the marker Jcuint057 located in *qC18:1-1*, showed the higher C18:1 content (43.0%) than Aa (30.9%). By contrast, progeny with Aa genotype at the marker Jatr872 located in *qOilC-4*, showed the higher total oil content (38.0%) than AA (33.7%) (Figure 3). These results suggested the effect of the two QTLs are opposite on these two key oil traits and favorite alleles were differentially from *J. curcas *and *J. integerrima*.

### eQTLs for oleosin genes

SNP markers were developed in *OleI*, *OleII *and *OleIII *genes (Table 4), which were mapped on LGs 5, 3 and 5 respectively (Figure 2).

**Table 4:** SNP markers and real time PCR primer pairs for *OleI*, *OleII *and *OleIII *genes

Gene	Forward primer (5'-3') Reverse primer (5'-3')	PCR product length (bp)	For SNP or Real time PCR use
*OleI*	CATTGCGCTAGCTGTTGCGACTCC	207	SNP and Real time PCR
	CGCCGCTTTGCCATTTCCATCT		
*OleII*	GGGGCTATGGGGCTCACAG	313	SNP and Real time PCR
	GTTGAGTTGGTTTATGGGGGATCT		
*OleIII*	ACAGCCACGATCCCACCAAGTAGT	443	SNP
	GGACAGAGCTGAGCAGTTTGGACA		
*OleIII*	TGGTGCCGACGGTTATCAC	216	Real time PCR
	TACATGCTGTCCAAACTGCTCAG		

*OleI *and *OleIII *were mapped on LG5 where the QTLs *qC18:0-5*, *qC18:1-5 *and *qOleIII *underlying C18:0, C18:1 and *OleIII *expression clustered. Negative additive effect value of *qOleIII-5 *indicated that *J. curcas *alleles were positive for *OleIII *expressions, of which LOD score was 3.1. This eQTL of *OleIII *was localized near *OleIII *gene and overlapped with the QTLs controlling C18:0 and C18:1, revealing a cis- or trans-element for *OleIII *which subsequently controlling the C18:0 and C18:1.

One eQTL on LG8 *qOleI-8 *was detected underlying *OleI *expression with LOD 1.9 (Table 3; Figures 1 and 2). Additive effect value of *qOleI-8 *was positive, indicating that *J. integerrima *alleles were positive for *OleI *expressions. To find as many putative QTLs (eQTLs) as possible, and to obtain a clearer understanding of the relationships among examined traits, a threshold eQTL of 1.9 for declaring a suggestive eQTL was employed. Low thresholds may not be useful in plant breeding programs but they have been shown to help in understanding relationships among traits [18].

*OleII *was located on LG3. One eQTL for *OleII *was detected on LG6 with LOD 2.6, which closed to *qC18:0-6*. It is suggested that a trans-element for *OleII *could harbor in this region which controlling the C18:0. Additive effect values indicated that *J. curcas *alleles were negative, indicating that the effect of *J. curcas *alleles was positive for *OleII *expressions.

## Discussion

### Development of inter-specific populations

To broaden the genetic diversity of cultivated crops and to identify QTLs associated with beneficial traits, such as yield, grain quality and disease resistance, development of inter-specific populations is a feasible strategy[19]. We developed around 500 SSR markers in jatropha, but very low polymorphism was detected within *J. curcas*, indicating the genetic variation was very limited within *J. curcas*. Thereby, we successful constructed a QTL/eQTL mapping population by crossing *J. curcas *to *J. integerrima *and generating a backcrossing population, and observed enhanced genetic diversity on DNA, RNA and phenotype levels, which was the prerequisite for QTL and eQTL detection.

For oil trait improvement, the interspecific hybridization approach is also viewed as a viable method to introgress the traits of interest, i.e. namely more liquid olein in oil palm [20]. With MAS, selection can be carried out in segregating generations of interspecific hybrids and their backcrosses more discriminately using molecular markers linked to the specific fatty acids. We investigated effects of the QTLs on oil traits and found that favorite alleles were originated from not only *J. curcas *but also *J. integerrima*. C18:1 in *J. curcas *was higher than in *J. integerrima*, while total oil content in *J. integerrima *was 51.04%, much higher than in *J. curcas *(Table 1). Consistent to this result, *qC18:1-1 *and *qOilC-4*, controlling C18:1 and total oil content respectively, were detected with the favorite alleles originated from *J. curcas *and *J. integerrima *respectively. Therefore, the QTL mapping population will be very useful for transferring favorite alleles form the two parents by further backcrossing and marker assisted selection.

Various germplasms were successfully utilized for development of chromosome segment substitution lines for studies on pest and disease resistance and other agronomic triats in rice [21-23]. Here we generated backcross populations for map construction and QTL mapping, which required less time to be developed and being 'immortal' for future QTL mapping due to jatropha's perennial life cycle. Meanwhile, the specific advantage of backcross populations is that, the populations can be further utilized to develop chromosome segment substitution lines for marker-assisted backcross breeding. The chromosome segment substitution lines will provide a valuable tool for jatropha germplasm enhancement, and can be expected to reveal the genetic basis of traits specific to the donor *J. integerrima*.

### Linkage or pleiotropic effect of genes in QTL cluster

Several chromosomal regions were associated with more than two traits indicating either linkage or pleiotropic effect. We detected a QTL cluster controlling C18:1 and C18:2 contents on the same region, i.e. closed to marker *Jcuint057 *on LG1 and Jcuint180 on LG10 with the additive value of C18:1 opposite to that of C18:2. This could explain the strong negative correlation between C18:1 and C18:2 (Table 2), which was consistent to the fact that linoleic acid is desaturated from oleic acid. Especially on LG1, the QTL was detected with a highly significant effect, accounting for 36.0% of the variation. It is revealed that either certain genes coexisted in these QTLs or a certain gene with pleiotropic effect in fatty acid metabolism pathway by modulating both C18:1 and C18:2 contents simultaneously. It will be meaningful to conduct fine mapping of these QTLs, isolate the target genes, and understand whether linkage or pleiotropic effect. The QTL regions were still distant to the flanking markers with linkage distance larger than 2 cM. Fine mapped QTL will speed up genetic improvement through MAS [3]. Construction of a high-resolution genetic linkage map of jatropha is underway, which will lay a solid foundation for a variety of future genetic and genomic studies, including QTL fine mapping and marker assisted selection.

### eQTL analysis of oleosin genes

To examine the function and modulation of oleosin genes in jatropha, we determined the expression levels of *OleI*, *OleII *and *OleIII *in the QTL mapping population, and conducted analysis with an approach named "genetical genomics" for identifying the genomic regions influencing gene expression [12,24]. The correlation of a structural gene's map position and its eQTL provides an indication of its regulation [24]. If the position of one gene and its eQTL are congruent, cis-regulation could be inferred, which means that the allelic polymorphism of the gene itself, or closely linked regulatory elements, directly impact the gene's expression. In this study, the eQTL for oleosin genes do not colocalize with these gene. This result suggests that the observed differences in oleosin gene expressions could be the consequences of trans-regulation, which means that gene expression is mainly regulated by trans-acting factors. A similar phenomenon has been observed for a set of genes involved in the biosynthesis of lignin in Eucalyptus. Most of these genes were significantly influenced by two eQTLs on LGs 4 and 9, whereas the structural genes were distributed throughout the entire genome [25].

The significant but low correlations were observed between oil traits and the expressions of oleosin genes. Similar phenomenon was reported by Yin et al [10]. They reported that the significant correlation between the expression of both GmRCAa and GmRCAb and Rubisco initial activity, photosynthetic rate, and seed yield indicated that these genes could play a role in increasing photosynthetic capacity and seed yield. However, the correlation coefficients between gene expression and Rubisco initial activity, photosynthetic rate, and seed yield were relatively small. This was also reflected by the fact that no coincident QTL (eQTL) was found between gene expression levels and the other three traits. Thus, they concluded that factors other than GmRCAa and GmRCAb limited photosynthetic capacity and seed yield. In our study, significant but low correlations between oil traits and the expressions of oleosin genes indicated that these genes could affect fatty acid composition and content; meanwhile, there should be other complex factors together with oleosin genes affecting oil traits.

The three eQTLs will provide possible approaches to oil trait improvement beyond previous QTL mapping results. Interestingly, *OleIII *gene, eQTL of *OleIII qOleIII-5 *and QTL of *qC18:0-5 *and *qC18:1-5 *were clustered on the same region on LG5. To further address whether a cis- or trans-element for *OleIII *harbored on LG5 subsequently controls the fatty acid compositions, fine mapping the two loci is still needed. Only eQTL of *OleIII *was coincident with QTL for oil composition, this result could be resulted from function differentiation of oleosin genes.

## Conclusions

In conclusion, we identified 18 QTLs underlying the oil traits and 3 eQTLs of the oleosin acid genes. Among them, *qC18:1-1*, *qOilC-4 *and *qOleIII-5*, controlling oleic acid, total oil content and oleosin gene expression respectively, were detected with relatively high contribution rates (R^2^) and could be expected to be applied in MAS by integrating more markers in these region. These data represents the first successful detection of QTLs/eQTLs underlying key agronomic traits in jatropha.

## Methods

### Plant material and plant growth conditions

*J. curcas *PZM16 was crossed to *J. integerima *S001 and hybrids CI7041 were generated. Then a backcrossing (BC) population was constructed consisting 286 individuals derived from the backcross PZM16 × CI7041. The population and parental lines were planted under standard growth conditions in experimental field of Lim Chu Kang farm, Singapore.

### Isolation of genomic DNA and synthesis of cDNA

Total DNA from leaves was extracted and purified using the DNeasy plant mini kit (QIAGEN, Germany). Oil bodies are located inside the cells of mature seeds. Total oil content and fatty acid composition in mature seeds are agronomic traits of importance. To investigate expressions of oleosin genes in mature seeds which are used for oil extraction, total RNA was isolated from mature seeds using plant RNA purification reagent (Invitrogen). Poly(A) tails were then added to the 3' end of the RNAs by poly(A) polymerase (Ambion), and the polyadenylated RNAs were reverse transcribed by SuperScript II reverse transcriptase (Invitrogen) with the oligo(dT) 3'-RACE adaptor (Ambion).

### Trait measurement and data collection

Each sample of QTL mapping population was grinded with liquid nitrogen, divided into 3 copies. Every sample consists of 3 mature seeds collected randomly from the same tree. Fatty acid compositions were analyzed by Gas chromatography (GC). Total lipid, extracted from 100 mg mature seeds, was transmethylated with 3 N methanolic-HCl (Sigma, St. Louis, MO, USA) plus 400 μL 2,2,-dimethoxypropane (Sigma, St. Louis, MO, USA). Oil was extracted using solvent (hexane) extraction followed by esterification to transfer from oil to methyl ester. The fatty acid methyl esters (FAME) was analyzed by GC using GC Agilent 6890 (Palo Alto, CA, USA) employing helium as the carrier gas and DB-23 columns for components separation. The GC analytical method was performed at 140°C for 50 s and a 30°C min^-1 ^ramp to 240°C, and the final temperature was maintained for 50 s for a total run time of 32 min. FA composition value included in the analyses was calculated based on peak area.

To amplify the mRNA from the reverse transcribed cDNAs and determine expression levels, real-time PCR was conducted with Real-Time PCR machine (I-Cycle, BioRad). Each reaction contained 200 ng of first-strand cDNAs, 0.5 μL of 10 mmol L^-1 ^gene-specific primers, and 12.5 μL of real-time PCR SYBR MIX (iQ™ SYBR^® ^Green Supermix, Bio-Rad). Amplification conditions were 95°C for 5 min followed by 40 cycles of 95°C for 15 s and 60°C for 60 s. The jatropha 18S rRNA was selected as the endogenous reference was used as a control to test for sample-to-sample variation in the amount of cDNA. cDNA from mature seeds of jatropha PZM16 was used as the calibrator on each real-time PCR plate. Two technical replicates of each reaction were performed. Normalized expression for each line was calculated as described in [10], i.e. ΔΔC_T _= (C_T, Target _- C_T, 18S_) _genotype _- (C_T, Target - _C_T, 18S_)_calibrator_. Lower ΔΔC_T _value means stronger gene expression and vice versa. Five mature seeds from each plant of QTL mapping population were used to determine the relative expression levels of *OleI*, *OleII *and *OleIII*. The results presented are means of the biological replicates for each plant.

### DNA markers and genotyping

Ninety-five markers almost evenly covering the 11 LGs were selected from a first-generation linkage map of jatropha [13]. One primer of each pair was labeled with FAM or HEX fluorescent dyes at the 5'end. The PCR program for microsatellite amplifications on PTC-100 PCR machines (MJ Research, CA, USA) consisted of the following steps: 94°C for 2 min followed by 37 cycles of 94°C for 30 s, 55°C for 30 s and 72°C for 45 s, then a final step of 72°C for 5 min. Each PCR reaction consisted of 1× PCR buffer (Finnzymes, Espoo, Finland) with 1.5 mM MgCl_2_, 200 nM of each PCR primer, 50 μM of each dNTP, 10 ng genomic DNA and one unit of DNA-polymerase (Finnzymes, Espoo, Finland). Products were analyzed using a DNA sequencer ABI3730xl, and fragment sizes were determined against the size standard ROX-500 (Applied Biosystems, CA, USA) with software GeneMapper V3.5 (Applied Biosystems, CA, USA) as described previously [26].

### Statistical analysis and QTL (eQTL) mapping

QTL (eQTL) analysis allows the genetic basis of variation of quantitative traits of interest to be dissected. Scoring every individual of a mapping population for the trait of interest and establishing a genetic linkage map for that population are two prerequisites for QTL (eQTL) detection. In this study, expression level data of fatty acid composition and content, and *OleI*, *OleII *and *OleIII *expression levels of the backcross population consisting of 286 individuals were collected with 3 replications. Pearson phenotypic correlations among traits were calculated by SAS PROC CORR. The 95 markers were genotyped in the QTL mapping population. SNP markers for mapping the three genes and primer pairs for determining expression levels by real-time PCR were listed in Table 4.

Linkage map was constructed using the software CRIMAP 3.0 to detect linkage and build map [27]. All multipoint distances were calculated using the Kosambi function. MapChart 2.2 software was used for graphical visualization of the linkage groups [28]. QTL (eQTL) analysis was performed using QTL Cartographer version 2.5 [29]. Model 6 of composite interval mapping was deployed for mapping QTLs (eQTLs) and estimating their effects. The genome was scanned at 2 cM intervals, and the forward regression method was selected. The log of the odds (LOD) score for declaring a significant QTL (eQTL) by permutation test analyses (1,000 permutations, 5% overall error level) as described previously. To find as many putative QTLs (eQTLs) as possible, and to obtain a clearer understanding of the relationships among examined traits, a threshold eQTL analysis of oleosin genes in of 2.0 for declaring a QTL (eQTL) was employed. Low thresholds may not be useful in plant breeding programs but they have been shown to help in understanding relationships among traits [18].

The maximum LOD score along the interval was taken as the position of the QTL (eQTL), and the region in the LOD score within 1 LOD unit of maximum was taken as the confidence interval. Additive effects of QTL (eQTL) detected were estimated from composite interval mapping results as the mean effect of replacing hybrid (CI7041)'s alleles at the locus of interest by *J. curcas *(PZM16) alleles. Thus, at a QTL (eQTL) having a positive effect, the alleles of *J. curcas *will increase the trait value. The contribution of each identified QTL (eQTL) to total phenotypic variance (r^2^) was estimated by variance component analysis. QTL (eQTL) nomenclature was adapted as following: starting with "q," followed by an abbreviation of the trait name, the name of the linkage group and the number of QTL (eQTL) affecting the trait on the linkage group.

In order to investigate associations between phenotypic traits and genotypes of two QTLs on LGs 1 and 4, mean phenotypic values of traits were calculated for those progeny with the alternate alleles of the microsatellite markers, inherited from the *J. integerrima *(aa), alleles inherited from the *J. curcas *(AA). A two-way ANOVA was performed on the progeny using two allelic combinations (AA, Aa) from markers linked to QTLs. This was conducted by using the general linear model (GLM) procedure of SAS (SAS Institute) and the Bonferroni method of multiple comparisons with α < 0.01.

## Authors' contributions

PL and CMW performed the experiments for collecting genotype and phenotype data. CMW designed the experiments, analyzed the data and drafted the manuscript. GHY supervised the project on jatropha molecular breeding and revised the manuscript. LL measured the oil traits; FS extracted DNA and RNA of the QTL mapping population; FS and PL participated in laboratory and field work for data collection. All authors read and approved the final manuscript.
